# Supplementary material for: Mitochondrial double-stranded RNA accumulation in brain aging and Alzheimer’s disease
Source: bioRxiv. 2026 Feb 4:2026.02.02.703345. Preprint. [Version 1] doi: 10.64898/2026.02.02.703345 (PMC12889667; doi:10.64898/2026.02.02.703345)
Supplement: Supplement 1 [file media-1.pdf]

SUPPLEMENTAL INFORMATION

Evidence of a role for mitochondrial double-stranded RNA accumulation in brain aging and Alzheimer's disease

Rachel Doser[1,2] and Thomas LaRocca[1,2]

Table S1:

NABEC Dataset Subject Characteristics			
**Age Group**	**n =**	**Sex**	**Age**
<35	26	62% M / 38% F	30.00 ± 3.84
36-45	36	72% M / 28% F	41.19 ± 2.72
46-55	27	66% M / 33% F	49.74 ± 2.67
>56	16	81% M / 19% F	66.81 ± 9.48

Table S2:

ROSMAP Dataset Subject Characteristics								
**Group**	**n =**	**Sex**	**Age at death**	**Education (total yrs)**	**% with APOE e4**	**MMSE**	**Braak**	**Cerad**
NCI	72	75% M 25 % F	87.60 ± 4.15	16.06 ± 3.17	16.6%	27.64 ± 2.53	3.19 ± 1.56	2.57 ± 1.23
MCI	38	71% M 29% F	87.95 ± 3.34	15.10 ± 2.88	7.9%	24.97 ± 3.64 ***	3.52 ± 1.31	2.07 ± 0.99
AD	106	79% M 21% F	88.87 ± 2.45	15.34 ± 3.09	29.2%	12.06 ± 9.01 ***	4.34 ± 0.98 ***	1.66 ± 0.87 ***

Table S3:

ROSMAP Metadata Subset Subject Characteristics													
**Group**	**n =**	**Sex**	**Age at death**	**Educ. (total yrs)**	**MMSE**	**Braak**	**Cerad**	**Global Cognition**	**Episodic Memory**	**Semantic Memory**	**Working Memory**	**Percep. Orientation**	**Speed Percep.**
NCI	3	33% M 66 % F	All > 90	13.33 ± 1.15	28.00 ± 1.00	3.00 ± 0	3.00 ± 1.00	0.04 ± 0.16	0.28 ± 0.10	0.23 ± 0.52	0.04 ± 0.52	-0.11 ± 0.31	-0.46 ± 0.71
MCI	4	25% M 75% F	All > 90	11.5 ± 1.73	25.25 ± 3.20	3.50 ± 1.73	2.00 ± 0.81	-0.73 ± 0.64	-0.39 ± 0.37	-0.35 ± 0.76	-0.56 ± 0.24	-0.67 ± 0.49	-1.14 ± 1.14
AD	7	43% M 57% F	89.83 ± 0.30	14.42 ± 1.98	10.43 ± 8.71*	1.11 ± 0.09	2.00 ± 0.81	-1.58 ± 0.61*	-1.71 ± 0.91*	-1.49 ± 0.88*	-1.02 ± 0.52*	-0.73 ± 0.94	-1.91 ± 0.38*
